# Determination of the amount of leg length inequality that induces scoliotic spinal posture changes in healthy subjects using video rasterstereography

**DOI:** 10.1186/1748-7161-8-S2-O38

**Published:** 2013-09-18

**Authors:** Marcel Betsch, Michael Wild

**Affiliations:** 1 Oregon Health & Science University, Portland, OR USA

## Background

Leg length inequalities (LLIs) can result in a functional scoliosis, increased energy consumption, abnormal gait or osteoarthritis of the hip[1]. In a previous study we simulated different LLIs of up to 15 mm and evaluated their effects on the pelvic position and spinal posture[2]. We found a correlation between LLIs and resulting changes of the pelvic position. Despite suggestions in the literature, we were not able to detect significant changes of the spinal posture. 

## Purpose

The purpose of this study was to determine the amount of LLI that would alter the spinal posture and induce scoliotic changes of the spine. 

## Methods

All subjects (n=110) were placed on an adjustable height platform, which was precisely controlled by the measuring device to simulate different LLIs of up to 20 mm. For LLIs greater than 20 mm, additional precision-cut wooden blocks were used under one foot. After an adaptation period, the resulting changes of the pelvis and spine were measured with a rasterstereographic device. Unifactorial ANOVA was calculated to check for changes in the mean values. The level of significance was set at p < 0.05. 

## Results

We found a significant correlation between platform height changes and changes of the pelvic position. The frontal spinal parameters surface rotation and lateral deviation changed significantly when simulating differences greater than 20 mm. In addition, the results showed that the spine always deviated toward the short leg side No changes of the sagittal spinal curvature were measured; however, a trend toward decreasing kyphotic angles was noted

## Conclusions and discussion

Our study has shown that LLIs greater than 20 mm will lead to scoliotic changes in the spinal posture of healthy test subjects. However, these changes were only found in frontal (surface rotation and lateral flexion) spinal parameters, and not in sagittal parameters. For the kyphotic angle, only a tendency toward decreasing angles was noted. In addition, we found a significant correlation between different leg lengths and changes of the pelvic position. Further, females and males seem to react in the same ways to LLIs. 
